# Clinical Spectrum of Long COVID: Effects on Female Reproductive Health

**DOI:** 10.3390/v16071142

**Published:** 2024-07-16

**Authors:** Syeda Maham, Mee-Sup Yoon

**Affiliations:** 1 Department of Health Sciences and Technology, Gachon Advanced Institute for Health Sciences and Technology, Gachon University, Incheon 21999, Republic of Korea; syedamaham.bbt@gmail.com; 2 Department of Molecular Medicine, College of Medicine, Gachon University, Incheon 21999, Republic of Korea; 3 Lee Gil Ya Cancer and Diabetes Institute, Gachon University, Incheon 21999, Republic of Korea

**Keywords:** COVID-19, female reproductive health, menstrual irregularities, long COVID, ovarian reserve, sex-specific outcomes

## Abstract

The COVID-19 pandemic caused by SARS-CoV-2 has presented numerous health challenges, including long-term COVID, which affects female reproductive health. This review consolidates the current research on the impact of SARS-CoV-2 on the menstrual cycle, ovarian function, fertility, and overall gynecological health. This study emphasizes the role of angiotensin-converting enzyme receptors in viral entry and the subsequent tissue-specific pathological effects. It also explores the potential influence of long COVID on hormonal balance and immune responses, contributing to menstrual irregularities and impaired ovarian function. The findings indicate a higher prevalence of long-term COVID-19 among women, highlighting the substantial implications for reproductive health and the need for sex-sensitive longitudinal studies. Enhanced surveillance and targeted research are essential to develop effective interventions that prioritize women’s reproductive well-being following SARS-CoV-2 infection. This review advocates for a sex-informed approach to ongoing COVID-19 research and healthcare strategies, aiming to provide up-to-date and pertinent data for healthcare providers and the general public, ultimately improving outcomes for females affected by long COVID.

## 1. Introduction

The COVID-19 pandemic emerged in Wuhan, China in late 2019 and rapidly evolved into a global health crisis characterized primarily by severe respiratory symptoms, causing widespread concern [1]. As of October 2022, the pandemic has resulted in approximately 6.5 million deaths worldwide, with individuals with underlying conditions, such as diabetes, cardiovascular diseases, and compromised immune systems due to cancer being particularly vulnerable [2,3].

Individuals previously affected with COVID-19 may continue to experience persistent symptoms or develop new symptoms several months after their initial exposure to SARS-CoV-2. This condition, known as long COVID or post-acute COVID-19 syndrome, is recognized by the World Health Organization (WHO) and manifests in individuals with probable or confirmed SARS-CoV-2 infection within three months of COVID-19 onset [4]. In most individuals infected with SARS-CoV-2, the live virus is eliminated from the body within a few days to weeks after infection, making it undetectable in the respiratory tract. However, the removal of viral RNA or antigens from the respiratory tissues or other body areas can take longer [5]. Long COVID is characterized by lingering symptoms that cannot be explained by any other diagnosis [4].

A report from 2022 indicated that 6.9% of the adult population experienced long COVID at any given time, with 3.4% being currently affected [6]. Women exhibited a higher incidence of long COVID (8.5%) than men (5.2%). Furthermore, women are more likely to be affected by long COVID (4.4%) than men (2.3%) [7]. Sex-related disparities have significant implications for infection-related diseases and influence research priorities and our understanding of long COVID [8]. Reproductive health factors, including pubertal changes, pregnancy, and menopause, may potentially affect the course of the disease in women with conditions such as myalgic encephalomyelitis/chronic fatigue syndrome (ME/CFS) and postural orthostatic tachycardia syndrome (POTS), often resulting in dizziness and exacerbation of the preexisting symptoms [9].

Critically, emerging research suggests that long-term COVID disproportionately affects female patients, affecting their reproductive health, including menstrual cycles, ovarian function, and fertility [10]. This review highlights the current understanding of the virological mechanisms of SARS-CoV-2, particularly its interaction with ACE2 receptors, which are crucial for viral entry and widely expressed in reproductive tissues. These interactions may lead to hormonal imbalances and specific reproductive health disorders [11]. The review covers the broader implications of these findings, emphasizing the need for a sex-sensitive approach to ongoing research and healthcare responses to the pandemic. We aimed to provide a comprehensive overview of the virological, clinical, and sex-specific aspects of long COVID, contributing to a better understanding of its long-term impact on female reproductive health.

## 2. Symptoms, Impact on Daily Living, and Pathology of Long COVID

### 2.1. Impact and Prevalence of Long COVID on Daily Activities and Health

Long COVID, also known as post-acute COVID-19 condition (PCC), is a complex syndrome that occurs after the acute phase of infection [12], and it is characterized by diverse symptoms that can affect various organ systems and significantly affect a patient’s quality of life. These symptoms can persist for several months and vary in presentation and severity. The US Household Pulse Survey reported that 78.5% of adults with PCC experienced different activity limitations [13]. Similarly, the Public Health Agency of Canada discovered that 21.3% of individuals face restrictions in their daily activities, whereas the UK ONS reported a higher percentage (58.6%). Research suggests a significant decline in the performance of activities of daily living (ADLs) as measured by validated scales, such as the Barthel and Katz indices. This decline was particularly pronounced among patients previously hospitalized for COVID-19 [12].

### 2.2. Symptoms and Pathology of Long COVID

The classification and symptoms of long COVID exhibit extensive variations and can be categorized based on different bodily systems. The commonly reported symptoms include joint pain, fatigue, chest discomfort, shortness of breath, hair loss, chest pain, weight gain, anxiety, depression, and memory impairment. These symptoms affect various organ systems, including the respiratory, neurological, and cardiovascular systems [5,14]. Less common but severe symptoms include multisystem inflammatory syndrome, a significant condition that may arise [15]. The intensity and duration of the symptoms differed significantly among the patients. They are often associated with factors such as the severity of the initial infection, the patient’s immune response, and potential genetic factors [16].

**Figure 1:**
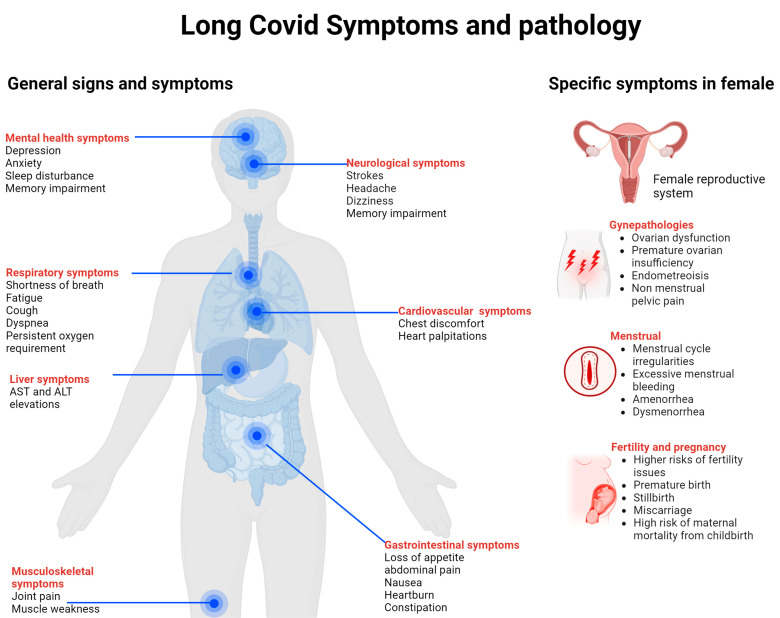
General and female-specific symptoms and pathology of long COVID.

The pathophysiology of long COVID involves immune dysregulation, cytokine storms, and coagulation abnormalities. Prolonged immune activation can result in the dysregulation of the immune response, leading to chronic inflammation and subsequent symptoms, such as “brain fog” and fatigue [17]. Additionally, immune responses can trigger abnormalities in blood coagulation, contributing to symptoms such as microclot formation, which can affect multiple organ systems [18] (Figure 1).

### 2.3. Mechanisms of SARS-CoV-2 Infection and the Persistence of Long COVID Symptoms

SARS-CoV-2 primarily enters the body through ACE-2 receptors and TMPRSS2 co-receptors on respiratory epithelial cells [19]. Moreover, it can infect other cell types in different organs, such as alveolar macrophages, renal tubular cells, gastrointestinal epithelia, esophageal keratinocytes, and liver cholangiocytes [20]. This ability to infect multiple systems explains the varied symptoms during acute SARS-CoV-2 infection and may contribute to prolonged symptoms in the long COVID [21].

In cases involving persistent symptoms, such as continuous anosmia or gastrointestinal disorders, it is possible to detect viral components, namely RNA and spike proteins. This method has the potential to elicit continuous immune responses [22]. These findings suggest that the ongoing symptoms of long COVID may be due to the immune system’s response to these residual viral elements rather than active viral replication. The continuous existence of antigens, especially in anatomical structures that are not readily accessible, poses a challenge to treatment and calls for the utilization of sophisticated detection techniques to gain a deeper understanding of and effectively manage long COVID.

### 2.4. Biomarkers in Severe Acute COVID-19 and Challenges in Long COVID Diagnosis

In severe acute COVID-19, the biomarkers primarily indicate systemic inflammation and damage to the endothelial cells. These biomarkers include increased levels of pro-inflammatory cytokines, chemokines, complement proteins, indicators of platelet activation. Additionally, the formation of neutrophil extracellular traps (NETs), and markers such as the D-dimer are elevated [23]. Combined with prolonged prothrombin time (PT) and activated partial thromboplastin time (APTT), these markers can suggest severe thrombocytopenia and disseminated intravascular coagulation (DIC) [24]. Lymphopenia, elevated levels of C-reactive protein (CRP), interleukins (IL-6 and IL-2R), lactate dehydrogenase (LDH), ferritin, markers of hepatic function, and troponins are associated with adverse outcomes such as acute myocardial infarction, venous thromboembolism, and stroke [25]. Unlike acute severe COVID-19, there are no specific diagnostic tests for long COVID because of its multifactorial nature. The biomarkers used in acute cases cannot be reliably attributed to long COVID, which has a wide range of clinical manifestations and requires personalized diagnostic assessments.

## 3. Gender Differences in Long COVID Manifestations

SARS-CoV-2 has the potential to affect women’s reproductive health primarily through its viral spike (S) protein. This protein binds to the ACE2 receptor and is processed by TMPRSS2 to enter the cells [26,27]. Additionally, the virus can enter cells through the BSG receptor and the CTSL protease [26,27]. An increased expression of these receptors in tissues increases the risk of infection. Although the presence of the virus has been detected to a limited extent in female reproductive secretions, such as vaginal fluids (5.7–12.5%) and cervical fluids (10.53%) [28,29], the direct assessment of SARS-CoV-2’s impact on reproductive organs, such as the endometrium and ovaries, is challenging owing to limitations in virus detection technology.

Recent research has shed light on the specific impacts of long COVID on different sexes. Women are more susceptible to long COVID, displaying a broader spectrum of symptoms than men [8]. Women tend to experience more severe symptoms, such as fatigue and neuropsychiatric issues, which persist for a longer duration compared to men. Furthermore, women are more prone to developing sexual dysfunction as a consequence of long COVID, which is often correlated with psychological stress and other symptoms associated with the condition [30,31]. Long COVID has a significant impact on women’s health, mainly affecting their reproductive health, psychological well-being, and overall quality of life. This condition is linked to various health issues, including disruption of the menstrual cycle, fertility concerns, and increased psychological stress.

Expanding on these observations, it is important to explore the various factors contributing to sex differences in long COVID outcomes, emphasizing the interplay between immune response, genetic predisposition, socio-cultural influences, and stress. Women typically exhibit a more vigorous innate immune response than men, leading to more severe symptoms during infections and stronger reactions to immunizations. This heightened immune reactivity may contribute to the increased prevalence and severity of long COVID symptoms in women [32]. Sex-linked genetic traits may influence susceptibility to long COVID. For example, genes on the X chromosome, such as TLR7 (involved in innate immunity), could contribute to sex-based differences in COVID-19 outcomes. Women, having two X chromosomes, may have an advantage in immune-related genes, but this could also predispose them to autoimmune-like responses seen in some long COVID cases [33]. Socio-cultural differences between sexes can impact health outcomes. Women are more likely to seek medical attention and report symptoms, leading to higher rates of long COVID diagnosis. Gender-based differences in occupational exposures, stress levels, and health behaviors may also influence long COVID risk and manifestation [34]. Stress significantly impacts both the hypothalamic-pituitary-adrenal (HPA) and hypothalamic-pituitary-gonadal (HPG) axes, which are already influenced by sex hormones. Chronic stress can lead to dysregulation of these axes, potentially exacerbating long COVID symptoms. Given the complex interactions between estrogen, cortisol, and immune function, women may be particularly susceptible to stress-induced alterations in these neuroendocrine pathways [35].

This figure illustrates the general signs and symptoms associated with long COVID, as well as specific symptoms observed in females. Commonly reported general symptoms include joint pain, fatigue, chest discomfort, shortness of breath, hair loss, chest pain, weight gain, anxiety, depression, and memory impairment. Additionally, female-specific symptoms involve pathologies related to the reproductive system, such as gynecological issues, menstrual irregularities, and pregnancy- and fertility-related concerns.

## 4. The Effect of Long COVID on Menstrual Irregularity

The menstrual cycle is a vital indicator of reproductive health and fertility, with irregularities potentially leading to complications, such as preeclampsia, low birth weight, and metabolic disorders [36,37]. Studies have shown that long COVID has a considerable impact on menstrual health, leading to a range of irregularities, including alterations in menstrual flow, cycle duration, and pain [9](Table 1). These findings illustrate the impact of long COVID on menstrual health, highlighting the need for more comprehensive studies to understand these associations better.

### 4.1. Mechanisms through Which SARS-CoV-2 Might Impact Menstrual Cycles

SARS-CoV-2 affects the menstrual cycle through both biological and psychological pathways. Infection has been associated with menstrual irregularities, which may arise from direct viral effects on reproductive tissues or indirectly through stress and inflammation [42]. One of the biological mechanisms involves the virus gaining entry into cells via the ACE2 receptor present in the ovaries. This infiltration can disrupt ovarian function and hormonal regulation, culminating in alterations in the menstrual regularity [43]. Another investigation demonstrated that fluctuations in the immune system across the menstrual cycle can influence susceptibility to infection, with a higher probability of irregularities occurring during the luteal phase [44].

### 4.2. Impact of the Renin–Angiotensin System on the Potential Effects of COVID-19

Angiotensin-converting enzyme (ACE) and ACE2 are pivotal in cardiovascular and reproductive physiology, as they facilitate the conversion of angiotensin I to angiotensin II and angiotensin II to angiotensin-(1–7), respectively [45]. These enzymes are integral to the regulation of blood pressure and fluid balance. Angiotensin II, a vital component of the renin-angiotensin system (RAS), increases vascular tone and blood pressure and plays a critical role in reproductive health. It enhances uterine blood flow and muscle contractility, is crucial for menstruation and embryo implantation, and stimulates aldosterone release from the adrenal cortex [46].

In contrast, Ang-(1–7) is associated with vasodilation and cardioprotection and supports the health of the endometrium and myometrium. ACE2 in the ovarian tissues renders them susceptible to SARS-CoV-2 infection, which can impair fertility by affecting follicular development and oocyte maturation [47]. The balance between Ang II and Ang-(1–7) is essential for hormone synthesis and oocyte maturation. COVID-19 may exacerbate oxidative stress, leading to chromosomal anomalies and potentially diminishing the efficacy of assisted reproductive technologies (ART). COVID-19 has been linked to reduced oocyte yield, particularly when oocyte retrieval is delayed after infection, indicating possible long-term effects on reproductive health [48].

Furthermore, the interaction between COVID-19 and ACE2 receptors within the female reproductive tract can reduce embryo implantation rates and quality. This suggests that long COVID might disrupt the RAS, affecting ovarian function, menstrual regularity, and the uterine environment, thereby impacting fertility and pregnancy outcomes [49]. These observations underscore the need for continued research on the long-term effects of COVID-19 on reproductive health and fertility, with a specific focus on the expression of ACE proteins in female-specific tissues.

### 4.3. Fluctuation of the Immune System during SARS-CoV-2 Infection

Fluctuations in the immune system during SARS-CoV-2 infection are influenced by hormonal changes throughout the menstrual cycle, particularly in progesterone and estrogen levels. During the luteal phase, elevated progesterone levels exhibit immunosuppressive effects, increasing the susceptibility to respiratory viruses, such as SARS-CoV-2. Progesterone promotes a Th-2-like cytokine profile and inhibits mast cell degranulation, creating a “window of vulnerability” post-ovulation [50].

Estrogen exerts protective effects by enhancing immune responses [51] and reducing the expression of the ACE2 receptor, through which SARS-CoV-2 enters cells. Additionally, it boosts the production of protective immune cells and antibodies [52]. The anti-inflammatory effects of estrogen help mitigate severe immune reactions, such as cytokine storms, which are characteristic of severe COVID-19 [53]. The protective role of estrogen highlights its potential therapeutic use in reducing COVID-19 severity and mortality [54].

Pro-inflammatory cytokines such as IL-6, TNF-α, and IL-1β can influence the production and function of sex hormones—particularly estrogen and progesterone—and these hormones, in turn, can modulate cytokine expression and activity [55]. Estrogen typically exhibits anti-inflammatory properties, but this protective effect may be disrupted during long COVID. For example, estrogen can suppress IL-6 and other pro-inflammatory cytokines, although this mechanism might be altered in COVID-19 [56]. Additionally, inflammatory cytokines can also suppress ovarian function, reducing estrogen production, which could diminish the anti-inflammatory response in women with long COVID [57]. It has been found that female COVID-19 patients had higher levels of innate immune cytokines, correlating with disease severity, which might contribute to increased long COVID symptom severity in women [58]. Evidence of hypothalamic–pituitary–gonadal axis dysfunction, including growth hormone and adrenal insufficiencies, has also been observed to be more prevalent in long COVID patients [59]. Furthermore, the influence of sex hormones influence on autoimmune diseases, are more common in women, suggests that hormonal fluctuations could exacerbate the potential autoimmune aspects of long COVID [60].

The interplay between estrogen and progesterone is crucial for modulating immune responses and influencing susceptibility to infections. Emerging studies have suggested that hormone replacement therapy (HRT) or selective estrogen receptor modulators (SERMs) can serve as adjunct treatments for COVID-19 [54], particularly in post-menopausal women with lower estrogen levels. Understanding the timing of hormone administration relative to the menstrual cycle can optimize therapeutic outcomes, minimize periods of heightened vulnerability, and enhance immune resilience. Additionally, the differential effects of estrogen and progesterone on immune function have significant implications for vaccine efficacy. The hormonal status may influence vaccine-induced immune responses, necessitating tailored vaccination strategies for women at different stages of their menstrual cycles or hormonal life [51]. Further research on the interaction between sex hormones and immune responses to vaccination could lead to more effective immunization protocols, especially for those at a higher risk of severe COVID-19.

In conclusion, the balance between estrogen and progesterone during the menstrual cycle is critical for modulating immune responses and susceptibility to infections, such as COVID-19. Recognizing these hormonal effects opens avenues for targeted therapies and personalized medical interventions, ultimately improving the management and outcomes of individuals affected by SARS-CoV-2.

## 5. Hormonal Influences and Reproductive Health Impacts of Long COVID in Women

The impact of long COVID on women’s reproductive health, particularly during puberty, pregnancy, and menopause, is a critical area of study. Changes in hormones, especially estrogen and progesterone, can affect the immune system and potentially influence the progression of SARS-CoV-2 infection via long COVID [9].

Infections can affect the hypothalamic–pituitary–gonadal (HPG) axis, leading to altered hormone production. For instance, it has been identified that SARS-CoV-2 can infect the testes, potentially disrupting testosterone production [61]. Similar mechanisms could affect ovarian function in women, altering the levels and anti-inflammatory effects of estrogen. Additionally, COVID-19 can affect thyroid function, which can influence sex hormone levels [62]. Thyroid dysfunction has been associated with menstrual irregularities and altered estrogen levels, potentially impacting the hormone’s anti-inflammatory properties. Severe COVID-19 can also lead to adrenal insufficiency [63]. The adrenal glands produce small amounts of estrogen, and their dysfunction could contribute to hormonal imbalances. Chronic inflammation can lead to a state of hormone resistance, including resistance to the anti-inflammatory effects of estrogen [55]. This mechanism could explain why some women with long COVID may not benefit from estrogen’s typical anti-inflammatory properties. Furthermore, inflammatory cytokines can suppress ovarian function, potentially reducing estrogen production [57]. This suppression could result in a diminished anti-inflammatory response in women with long COVID. These studies imply that inflammation caused by infections may affect glands that produce hormones, thereby changing the amount and potency of estrogen produced. The observed sex variations in the duration and severity of COVID-19 symptoms could be attributed to this disturbance.

There are multiple ways in which SARS-CoV-2 can affect adrenal function. The virus directly impacts the cells of adrenal tissues that express the ACE2 receptor, causing impairment in their function [63]. Normal function is disrupted by the strong cytokine storm associated with COVID-19, which leads to inflammation and damage to adrenal structures [64]. Additionally, due to the unique vascular structure of the glands, COVID-19-related coagulopathy may cause adrenal infarction or bleeding. Severe illness can result in relative adrenal insufficiency or critical illness-related corticosteroid insufficiency (CIRCI) due to cortisol overproduction. The virus can also interfere with the hypothalamic–pituitary–adrenal (HPA) axis, altering cortisol dynamics and leading to central adrenal insufficiency [65]. Lastly, the absence of lymphopenia in COVID-19 patients may indicate hypercortisolism given the observed changes in lymphocyte counts in SARS [63].

Long-term inflammation can lead to hormone resistance, especially to the anti-inflammatory properties of estrogen [55]. The release of pro-inflammatory cytokines, continuous activation of immune cells, and persistent inflammatory signaling characterize this chronic inflammatory state. Chronic inflammation also affects the expression and function of estrogen receptors, altering the ratio of estrogen receptor α to β, downregulating the receptor in target tissues, and changing the signaling pathways of the receptor [66]. Pro-inflammatory cytokines counteract the anti-inflammatory effects of estrogen, interfere with normal estrogen signaling, and alter the expression of enzymes involved in estrogen metabolism. Furthermore, chronic inflammation affects the intracellular metabolism of estrogen, resulting in metabolites with varying degrees of inflammatory consequences. The effects of estrogen on inflammation depend on the tissue and are influenced by the duration and concentration of exposure [67]. Chronic inflammation and estrogen resistance also impact other physiological systems, including the sympathetic nervous system, hypothalamic-pituitary-adrenal axis, and sensory nervous system. These interactions collectively reduce or alter the natural anti-inflammatory effects of estrogen, potentially exacerbating inflammatory diseases and causing hormone resistance [55].

Inflammatory cytokines disrupt ovarian function through multiple pathways. They block the action of follicle-stimulating hormone (FSH) and luteinizing hormone (LH) from the pituitary gland and decrease gonadotropin-releasing hormone (GnRH) from the hypothalamus, disrupting the hypothalamic–pituitary–gonadal (HPG) axis. Cytokines directly affect ovarian tissues by interfering with follicular growth steroidogenesis and causing cell death in granulosa cells, which is essential for follicle development and hormone synthesis [68]. They also modify the expression and function of estrogen receptors, influencing the responsiveness of ovarian cells to estrogen and affecting the anti-inflammatory responses mediated by estrogen. Cytokine-induced oxidative stress impairs follicular growth oocyte quality, damages DNA, and disrupts cellular function. Additionally, cytokines affect ovarian angiogenesis, impacting ovulation, corpus luteum formation, follicular development, and hormone synthesis [69]. Furthermore, cytokines stimulate immune cells to infiltrate ovarian tissues, creating a localized inflammatory microenvironment detrimental to normal ovarian function. These pathways collectively contribute to reproductive failure in chronic inflammatory diseases such as COVID-19 or Long COVID [57].

The interaction between hormonal fluctuations and immune responses is significant during the different reproductive stages of COVID-19 [70]. Estrogen and progesterone can have pro-inflammatory or anti-inflammatory effects depending on their levels during the menstrual cycle. Post-COVID-19, a few individuals may experience irregular ovarian function due to excessive immunological or inflammatory responses. This response can disrupt the hypothalamic–pituitary–ovarian (HPO) axis, which is essential in the menstrual cycle [71,72,73,74]. Interactions between the hypothalamic–pituitary–adrenal (HPA) and hypothalamic-pituitary-gonadal (HPG) axes are crucial in menstrual regulation. The stress-induced disruption of gonadotropin secretion can hinder reproduction [75,76,77]. Although progesterone-only contraception may increase the menstrual flow [78], hormonal contraception has been suggested to mitigate post-vaccination menstrual changes [77,79]. Stress through the HPA axis may disrupt the menstrual cycle during the COVID-19 pandemic, linking cytokine-mediated stress to temporary menstrual disruption.

Another concern is the increase in precocious puberty associated with lifestyle changes during the lockdown. Increased sedentary behavior may enhance GnRH secretion, complicating long COVID during puberty. Significant immunological adaptations during pregnancy may affect susceptibility to and experience with long COVID [80]. Pregnant women with COVID-19 are at a higher risk of severe outcomes and prolonged post-recovery complications. Furthermore, post-menopausal women may experience unique long COVID symptoms due to decreased estrogen and progesterone levels, affecting immune regulation and increasing susceptibility to infections. Post-menopausal women are at a higher risk of severe COVID-19 outcomes owing to reduced sex hormone levels [81].

Moreover, hormonal imbalances have been reported over long COVID symptoms. Studies on the hormonal levels in 268 individuals showed significantly lower cortisol levels in those with long COVID, suggesting the pivotal role of cortisol in symptom persistence [82]. This hormonal imbalance may contribute to a prolonged recovery in a few women, marking an area for further investigation and development of targeted treatment strategies [83].

The intersection of reproductive health stages with long COVID provides a complex clinical scenario requiring focused research and targeted care approaches. Understanding how different reproductive stages influence the trajectory of long COVID is crucial for developing effective interventions and ensuring holistic, personalized care for women. Comprehensive care models incorporating psychological support, physical rehabilitation, and specialized reproductive health services are essential to addressing the unique needs of women with long COVID [84].

The impact of SARS-CoV-2 on female reproductive health significantly affects cellular and molecular processes. Specifically, it can adversely affect ovarian tissue and oocyte quality, potentially disrupting ovulatory function and resulting in aneuploid oocytes with a reduced likelihood of successful fertilization [85]. Follicular ovarian cells were co-cultured with SARS-CoV-2 and analyzed using qPCR, immunofluorescence, western blotting, and transmission electron microscopy, revealing significant viral activity and the presence of host factors essential for viral entry and replication [85]. Viral RNA, spike, and nucleocapsid proteins were detected in the granulosa and cumulus cells at multiple time points post-infection, confirming active viral replication [85]. Additionally, the cell culture supernatant successfully infected VERO E6 cells, and transmission electron microscopy showed virions in the cytoplasm and on the cell membrane, indicating the potential of COVID-19 to affect female reproductive health [86]. Although the virus does not directly infect the female reproductive system, it can indirectly influence sex hormone concentrations by triggering cytokine storms and inducing stress responses. In turn, this can affect menstrual and hormonal profiles [85]. However, a few studies have shown that SARS-CoV-2 does not significantly impair the ovarian response to controlled ovarian stimulation or embryo development following infection or vaccination [86].

## 6. Future Directions for Research on Long COVID and Women’s Health

Understanding the relationship between women’s health and long COVID is crucial for developing interventions that consider sex differences. The domains of reproductive health and hormonal influences present promising avenues for researching the complexities of the impact of long COVID on women. Longitudinal cohort studies are necessary to monitor hormone levels in women with long COVID. Participants should receive regular monitoring to assess hormonal changes in estrogen, progesterone, and cortisol related to the menstrual cycle and stress responses. This can help establish correlations between hormonal fluctuations and the severity or variation of long COVID symptoms over time. In addition, controlled clinical trials are required to introduce interventions that may stabilize hormonal fluctuations, such as hormone replacement therapy (HRT) or other hormonal modulators, and to measure their effects on long-term COVID outcomes. This approach can help determine whether stabilizing hormonal levels can alleviate the symptoms of long COVID.

Furthermore, cross-sectional studies can compare hormone levels and symptom profiles between women with long COVID and those without across different stages of their reproductive life (e.g., pre-menopause and menopause). This will help identify whether hormonal fluctuations have a differential impact on the reproductive stage. However, biomechanical studies have focused on the mechanistic pathways by which hormones affect cellular and molecular processes in long COVID. For example, studying the impact of estrogen on immune cell function or the role of progesterone in inflammation and tissue repair could provide insights into the pathophysiology of long COVID. Additional exploration is essential to reveal the mechanisms by which genetic and epigenetic factors influence hormonal regulation and responses in long COVID patients. This may include studies on gene expression related to hormone receptors or enzymes involved in hormone metabolism.

These studies will provide a comprehensive understanding of how hormonal fluctuations affect women with long COVID, supporting the development of targeted therapies and improving management strategies. Continued research in these areas is vital for developing a comprehensive understanding of the impact of long COVID on women’s health. Such efforts are expected to yield practical, equitable, and sex-sensitive treatment and support strategies, enhancing the quality of life of women affected by long COVID globally.

In conclusion, this review highlights the complex interactions among SARS-CoV-2 infection, long COVID, and female reproductive health. Although evidence suggests that infection and vaccination may cause temporary menstrual irregularities, their long-term effects on fertility and ovarian function are minimal. However, ongoing studies are essential given the dynamic nature of COVID-19 research. As the pandemic progresses, sustained vigilance and continuous research are vital to provide accurate and updated information to healthcare professionals and the public.

## 7. Conclusions

An urgent need exists for sex-sensitive longitudinal studies to develop effective interventions that prioritize women’s reproductive health. The findings of this review indicate a substantial impact of long COVID on female reproductive health, with menstrual irregularities, impaired ovarian function, and fertility concerns being prominent. These findings are consistent with those of other studies, highlighting the role of ACE2 receptors in reproductive tissues and the potential hormonal imbalances caused by SARS-CoV-2. This review highlights the need for sex-sensitive approaches in research and clinical practice. Future research should focus on longitudinal studies to monitor the long-term effects of COVID-19 on women’s reproductive health and develop targeted therapeutic strategies.

## Figures and Tables

**Table 1:** Overview of studies on menstrual changes associated with long COVID.

ID	Study Type	Sample Size	Main Findings	Citation
1	Cross-sectional study	n = 1792	Over one-third of menstruating long COVID patients reported worsening of symptoms the week before or during menses.	[38]
2	Cross-sectional study	n = 460	62% of long COVID patients experienced worsening symptoms days before menses.	[39]
3	Multi-country patient-led survey	n = 1792	33.8% reported menstrual issues, including abnormally irregular cycles (26%) and heavy periods (19.7%); 4.5% of women aged 49+ reported post-menopausal bleeding.	[38]
4	Survey study comparing to general population	n = 748 LC, n = 2299 with COVID-19 history, n = 15,156 without COVID-19 history	Long COVID patients reported higher rates of menstrual cycle changes (OR 1.34, 95% CI 1.15–1.57) compared to the general population with and without a history of COVID-19.	[40]
5	Longitudinal prospective cohort study	Not specified	16% of women and nonbinary people experienced menstrual cycle changes 28 to 222 days after SARS-CoV-2 infection, including irregular menstruation and increased PMS.	[41]
6	Retrospective case-control study	n = 1066 COVID-19 cases, n = 4989 vaccination	COVID-19, but not vaccination, was associated with an increased risk of changes in menstrual cycle duration, intermenstrual bleeding, increased flow, and missed periods.	[19]
